# Human, economic, and social impact of lysosomal storage diseases

**DOI:** 10.1186/s13023-025-04053-z

**Published:** 2025-11-25

**Authors:** Eduardo Brignani, María José de Castro-López, Antonio Gonzalez-Meneses, Montserrat Morales, Guillem Pintos-Morell, José Luis Poveda, Imma Vilalta, Jordi Cruz, Gabriel Fernando Ballester-Lozano, Héctor David de Paz

**Affiliations:** 1 Federación Española de Enfermedades Raras, Barcelona, Spain; 2 Manchester University NHS Foundation Trust, Manchester, UK; 3 Hospital Universitario Virgen del Rocío, Seville, Spain; 4 Hospital Universitario 12 de Octubre, Madrid, Spain; 5 Vall d’Hebron Institut de Recerca (VHIR), Barcelona, Spain; 6 Hospital Universitario y Politécnico La Fe, Valencia, Spain; 7 Hospital Sant Joan de Déu, Barcelona, Spain; 8 Asociación MPS-Lisosomales, Avda. Barcelona nº 174, 1º 2a, Igualada, Barcelona, 08700 Spain; 9 Outcomes Research Department, Outcomes’10, S.L., Castellón de la Plana, Spain

**Keywords:** Lysosomal storage disorders, Economic impact, Quality of life, Healthcare costs, Patient burden, Caregiver burden

## Abstract

**Background:**

Lysosomal storage disorders (LSDs) are a group of rare metabolic conditions caused by enzyme deficiencies, leading to the accumulation of macromolecules within lysosomes. These disorders significantly impact patients’ quality of life (QoL) and impose substantial financial burdens on families and healthcare systems. This study aimed to evaluate the daily life impact and economic burden of LSDs on patients, caregivers, the Spanish Health System (SHS), and society.

**Methods:**

This cross-sectional study used an ad hoc questionnaire targeting Spanish LSD patients and their caregivers. The questionnaire was based on a literature review and insights from patients, caregivers, and healthcare professionals (HCPs). Data were collected on sociodemographic characteristics, clinical variables, QoL, and economic costs. Caregivers provided responses on behalf of both themselves and the patients. Direct and indirect costs were assessed based on healthcare resource utilisation, productivity losses, and patient-incurred expenses.

**Results:**

The study included data on 86 patients, with 22 patients responding on their own behalf and 64 caregivers responding on behalf of themselves and their patient. Twelve different LSDs were identified, with Sanfilippo (22.1%) and Fabry (18.1%) being the most prevalent. The mean age at diagnosis was 9.9 years, with an average diagnostic delay of 4.3 years. Patients required an average of 107.8 medical visits per year, many of which were out-of-pocket, particularly for physiotherapy (28.6 visits/year) and psychological services. The total annual cost per patient was €228,232.60, with direct costs to the SHS accounting for 81.4% of this amount. Indirect costs, primarily due to informal caregiving and productivity losses, represented 15.9%. Families bore an average annual cost of €6,170.20, mainly for formal care and non-covered medical expenses. Clinically, 29.1% of patients had severe-to-profound functional limitations, and 31.4% had cognitive limitations, significantly affecting their daily activities, mobility, and emotional well-being.

**Conclusions:**

LSDs impose a substantial economic and social burden on patients and their families, with high healthcare costs and significant productivity losses. The study underscores the need for comprehensive support strategies addressing both economic and social challenges faced by affected families. Future research should explore country-specific economic impacts and develop policies to mitigate the financial strain on these families.

**Supplementary information:**

The online version contains supplementary material available at 10.1186/s13023-025-04053-z.

## Background

Lysosomal storage diseases (LSDs) are a group of metabolic disorders caused by lysosomal enzyme deficiencies and characterized by the accumulation of different macromolecules inside lysosomes, which leads to cell dysfunction and cell death [1]. LSDs comprise over 70 disorders caused by mutations in genes encoding lysosomal hydrolases. Based on the accumulated molecule, LSDs are classified into sphingolipidoses, mucopolysaccharidoses, glycogenoses, glycoproteinoses, and ceroid lipofuscinoses [1, 2].

Although epidemiology studies are scarce, LSDs are estimated to affect approximately 1 in 5,000 births. However, individual disorders are considered rare, with estimated prevalences ranging from approximately 0.03 to 7 in 100,000 births in different settings [3–5].

The age of onset can range from infancy to adulthood with patients with LSDs often presenting visceral (hepatosplenomegaly), ocular, haematological, skeletal, and neurological manifestations [6]. Overall, LSDs progress and evolve during time, negatively impacting patients’ quality of life (QoL) [7–9]. Consequently, patients present limitations to develop daily and social activities, including their ability to work [9]. In this context, and considering the life-limiting effects of LSDs, these disorders also impact on different domains of caregivers’ QoL, including health and professional status [10, 11].

Currently, there is no pharmacological treatment for most LSDs. However, there are few therapeutic approaches for the treatment of some LSDs such as Gaucher, Fabry, or Pompe [12]. Among these, enzyme replacement therapy presents the widest application and is considered the gold standard for LSDs [13]. However, given the clinical differences between LSD types, other approaches such as haematopoietic stem cell transplantation, substrate reduction therapy, pharmacological chaperones, and, more recently, gene therapy are needed [14]. Some of these treatments are associated with an elevated economic cost [15, 16]. This, together with the high healthcare resource utilization [17], represent a great economic burden for the Spanish Health System (SHS), particularly given that all approved pharmacological treatments for LSDs are funded by the SHS.

Considering the above, we aimed to explore the impact of LSDs on the daily lives of patients and caregivers, to estimate the economic burden of the disease for the affected families, healthcare system and society.

## Methods

### Study design

This is a cross-sectional study developed in the following five stages: 1) literature review, 2) focus groups with patients and healthcare professionals (HCPs), 3) questionnaire design, 4) survey directed at patients and caregivers, and 5) cost analysis.

The study was led by a Scientific Committee composed of HCPs (*n* = 7) from different specialties (neuropaediatrics, paediatrics, orthopaedic surgery, internal medicine, psychology, and hospital pharmacy) and one patient representative.

The study was approved by the Ethics Committee of the Hospital Puerta de Hierro (Madrid) and developed according to the Helsinki Declaration [18] and the Guideline for Good Clinical Practice [19].

### Literature review

A literature review was carried out in the international database Pubmed/MEDLINE and grey literature (Google Scholar and clinical practice guidelines and consensuses from the main Spanish scientific societies) to identify the current evidence available on the burden of LSD in Spain (Supplementary Table S1). Systematic review, consensus, observational studies, and Spanish clinical practice guidelines published in English and Spanish were included. Clinical trials, letters, communications at meetings, and studies not conducted in European settings were excluded.

### Focus groups

The focus group is a qualitative methodology that allows participants to describe their perceptions, opinions, beliefs, and attitudes toward a particular topic. Although there is no standard number of participants for a focus group, a composition of 5–10 members is ideal [20].

The main goal of both focus groups (patients and HCPs) was to complement the information extracted from the literature review to develop the study questionnaire.

#### Focus group with patients

The patient focus group was composed of eight members (patients and caregivers). All adult (≥18 years old) participants with a diagnosis of any type of LSD (or their caregivers) were identified and invited to participate by the Spanish MPS-Lisosomales Association. Participants gave signed consent to participate.

During the focus group, information on participants’ experience regarding the impact and burden of the disease on their QoL, daily activities, and associated economic costs were collected.

#### Focus group with experts

The focus group was composed of the eight members of the Scientific Committee. Based on the information extracted from the literature review and the patient focus group, the experts identified key aspects for the management of patients with LSDs and healthcare resource utilization associated with diagnosis, treatment, and follow-up. Furthermore, the rates of use of different clinical tests and their frequency for the diagnosis and follow-up of the most represented LSDs were estimated according to the percentage of patients using each defined resource and the frequency of use.

### Questionnaire

The questionnaire, directed at patients and caregivers, was developed based on the literature review and insights from two focus groups, with the goal of obtaining a comprehensive picture of the characteristics of Spanish patients with LSDs. Patients answering the questionnaire responded from their own perspective, while caregivers responded from the patients’ perspective and their own. There was no patient overlap between the two groups of respondents. This approach allowed us to gather data on 86 patients, combining both self-reported and caregiver-reported information.

The questionnaire, comprising up to 52 questions for patients and 63 for caregivers, was divided into four sections: 1) Sociodemographic characteristics, 2) Clinical variables, 3) Quality of life, and 4) Economic costs Both versions of the questionnaire (patient and caregiver) are available as Supplementary Material.

### Study population

Adult patients with a diagnosis of any type of LSD or their caregivers were invited to participate in the study through the web page and social media of the Spanish MPS-Lisosomales Association.

### Cost analysis/estimation

#### Direct costs

Direct costs estimated from healthcare resource utilization associated with LSDs were identified from the responses to the questionnaire and HCPs’ experience and included visits to primary care, primary care nursing, and specialists related to LSD, visits to the emergency department, hospitalization (number and length of stay [days]), diagnostic and laboratory tests, treatments associated with LSDs, and weekly formal care (hours).

Unitary costs (euros, 2024) for each resource were obtained from the eHealth database [21] of the Council of Pharmaceutical Associations [22], applying the deductions established in Royal Decree-Law 8/2010 [23] and official government gazettes.

Costs associated with formal care and each resource were estimated by multiplying the number of times the resource was used.

Costs were calculated on an annual basis. Mean annual costs per patient were calculated across the full study sample, assigning a value of zero to patients who did not use a given resource. For those variables in which costs or resources were estimated for longer periods of time [surgery type (3 years), medical devices, home or vehicle adaptations (5 years)], their value was divided by the number of years in the considered period. Diagnostic test utilization was estimated by HCPs as average annual use per patient. The period for home or vehicle adaptations was calculated subtracting the mean age at diagnosis from patients’ mean age.

Costs associated with specific treatments for LSDs were estimated from the unitary cost and the posology described in the treatment data sheet. The dosage calculation considered the mean age of patients for each type of LSD, estimating weight based on this age group and adjusting for Z-score variations relative to the general population of the same age group (Fabry = 70.0 kg; Gaucher = 70.0 kg; Hurler = 47.3 kg; Hunter = 64.4 kg; Maroteaux-Lamy = 30.3 kg; and Sly = 45.0 kg) [24, 25]. When more than one treatment was available, the average cost of treatments was used.

For the cost associated with laboratory tests, the most frequent tests used for the diagnosis/follow-up of patients were identified. HCPs estimated the annual healthcare resource utilization for the five most prevalent LSDs in the present study (Sanfilippo, Fabry, Hurler, Morquio, and Hunter) considering the estimated percentage of patients undergoing such tests and the annual frequency.

#### Indirect costs

Indirect costs were extracted from the questionnaire results and included the number of weekly working/study hours lost by the patient (due to disease) and by the caregiver, and the number of informal care hours (caregiver).

Costs associated with productivity losses were estimated by multiplying the number of working hours lost by the cost per working hour according to the INE, the Spanish Statistical Office [26]. Costs associated with informal care were estimated by multiplying the number of weekly hours of care by the cost/hour of the service equivalent to the service financed by the public administration [27]. Costs associated with work and academic productivity were calculated assuming the average hourly wage of the INE [26] and an average of 30 weekly teaching hours, respectively.

#### Costs for patients

The costs assumed by the family unit were extracted from the questionnaire results and included formal care financed by the patient, treatment, rehabilitation, and visits to specialists not included in the SHS, medical equipment paid for by the patient, and adaptations required at home.

Costs associated with formal care paid for by the patient were estimated by multiplying the number of weekly care hours by the cost of the professional’s average wage/hour.

## Statistical analysis

To ensure the absence of duplicate entries, where both a patient and their caregiver may have completed the questionnaire, a thorough review of key variables including type of disease, age, and sex was conducted. In instances where duplicate data were identified, only the patient’s responses were retained for analysis. Upon validation of the dataset, no duplicate entries were detected.

For quantitative variables, centrality (mean, median) and dispersion (standard deviation [SD], quartiles, minimum, and maximum) measures were calculated. For qualitative variables, relative and absolute frequencies were calculated.

All statistical analyses were carried out using software package STATA v14.

## Results

### Participants’ sociodemographic characteristics

A total of 22 patients with LSDs and 64 caregivers from 14 Spanish autonomous communities completed the questionnaire. The sociodemographic characteristics of the patients were collected through self-reported data and caregiver-reported data, making for a total of 86 respondents, covering data for 86 individual patients.

Patients (*n* = 86) had a mean (SD) age of 23.1 (17.0) years and 48.8% were women, while caregivers (*n* = 64) had a mean (SD) age of 47.6 (8.9) years and 71.9% were women. Patients who completed the questionnaire themselves had a mean age of 43.0 (13.3) years, while those represented by caregivers had a mean age of 17.0 (13.4) years.

Most patients (82.6%) required some type of care or assistance, the caregivers being in most cases a relative (82.6%) or more than one (48.9%). In addition, 16.3% of the patients in the study had formal care. Of these, 57.1% was financed by the family.

Most patients (86.0%) had a recognized disability and approximately half (53.5%) had a degree of disability > 65%.

On the other hand, 15.1% of the patients were employed, working a mean (SD) of 35.6 (9.0) weekly hours, 47.7% were students, and 11.6% were not schooled. Regarding caregivers, most (59.4%) were employed, working a mean (SD) of 29.8 (14.9) weekly hours, and 28.1% were unemployed due to their caregiver role (Table 1).

**Table 1:** Employment and academic status of study participants

Employment/academic status	Women (n = 42)	Patients	n (%)	Women (n = 46)	Caregivers	n (%)
		Men (n = 44)	Total (n = 86)		Men (n = 18)	Total (n = 64)
Unemployed due to the LSD	4 (9.5)	2 (4.6)	6 (7.0)	16 (34.8)	2 (11.1)	18 (28.1)
Unemployed due to other reasons	1 (2.4)	0	1 (1.2)	1 (2.2)	0	1 (1.6)
Retired due to the LSD	8 (19.1)	6 (13.6)	14 (16.3)	1 (2.2)	1 (5.6)	2 (3.1)
Retired due to other reasons	1 (2.4)	6 (13.6)	1 (1.2)	2 (4.4)	2 (11.1)	4 (6.3)
Employed	4 (9.5)	6 (13.6)	10 (11.6)	22 (47.8)	13 (72.2)	35 (54.7)
Self-employed	2 (4.8)	16 (2.3)	3 (3.5)	3 (6.5)	0 (0.0)	3 (4.7)
Student	18 (42.9)	23 (52.3)	41 (47.7)	1 (2.2)	0 (0.0)	1 (1.6)
Not schooled	4 (9.5)	6 (13.6)	10 (11.6)	-	-	-

LSD: Lysosomal storage disorder

Most participants (75.6%) indicated that family income was lower than €3,000 per month, with a weighted mean annual income of €26,032.20.

### Clinical characteristics

A total of 12 different LSDs were identified in the study sample, Sanfilippo (*n* = 19; 22.1%) and Fabry (*n* = 16; 18.1%) being the most common, followed by Hurler (*n* = 13; 15.1%), Morquio (*n* = 13; 15.1%), Hunter (*n* = 12; 13.9%), mucolipidosis II, III, IV (*n* = 5; 5.8%), Sly (*n* = 3; 3.5%), and Gaucher, gangliosidosis GMI, Maroteaux-Lamy, Batten syndrome and alpha-mannosidosis (all *n* = 1; 1.2%).

The mean (SD) age at diagnosis was 9.9 (14.6) years, with a mean (SD) time from symptom onset to diagnosis of 4.3 (8.9) years.

**Fig. 1:**
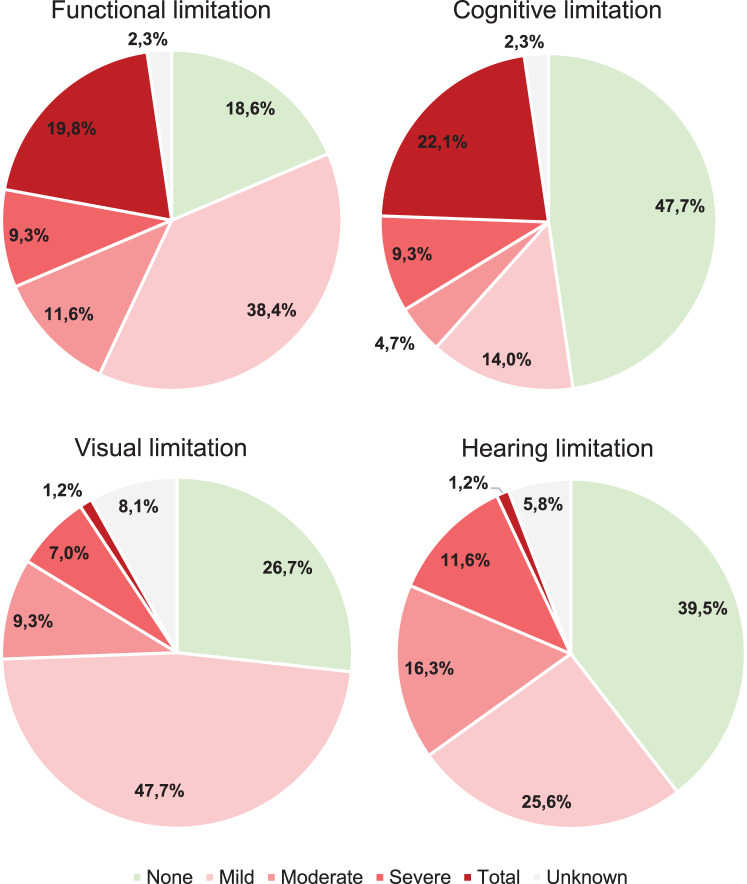
Type and degree of limitations

Overall, 29.1% and 31.4% of the patients with LSDs presented severe-to-profound functional and cognitive limitations, respectively (Fig. 1).

### Quality of life

**Fig. 2:**
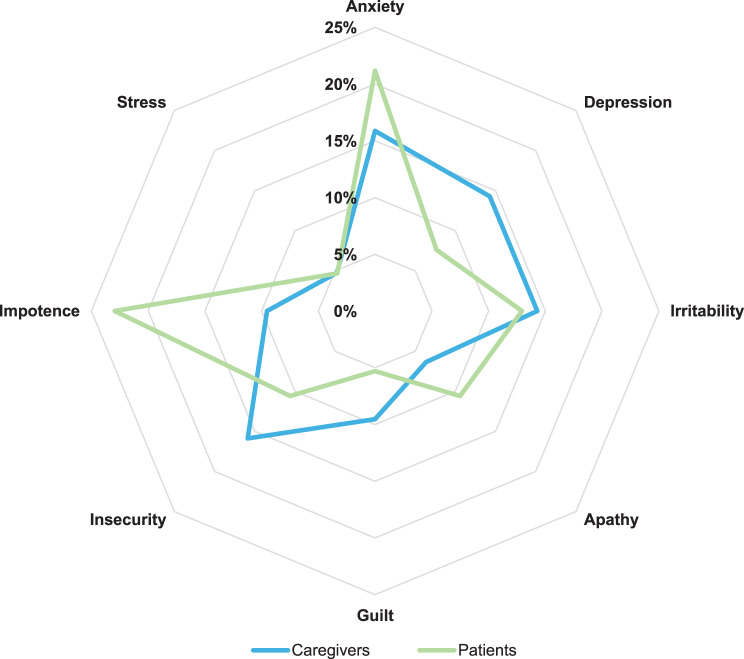
Emotional status of patients and caregivers

Participants rated the impact of the LSD on patients’ overall health status during the last month with a mean (SD) of 6.2 (2.3) points on a scale from 1 to 10. Taking only responses from adult patients into consideration, this group of participants also rated the impact of LSDs on intimate relationships, with a mean of 5.3 points. In addition, almost all patients (90.5%) and caregivers (95.9%) stated having mood swings during this same period of time, with anxiety, depression, insecurity, and irritability being the most frequent (14–15%) among patients (Fig. 2).

**Fig. 3:**
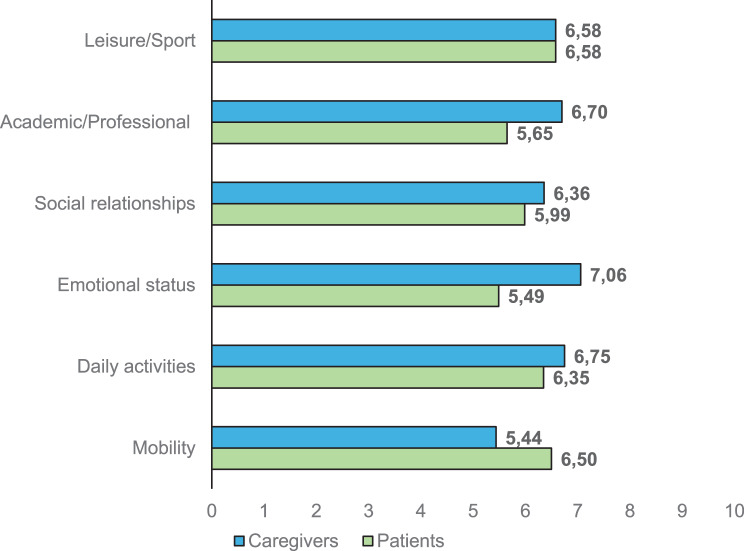
Impact of LSDs on different domains of quality of life and patients’ and caregivers’ overall health status

Regarding the impact of the disease, LSDs represent a moderate/high burden in different domains for both patients and caregivers. Patients reported the highest impact on their mobility, leisure or sport, and daily activities domains. Caregivers reported similar values for leisure and daily activities, with the emotional domain being the most affected (Fig. 3).

### Healthcare resource utilization

#### Medical visits

Patients required a mean of 107.8 medical visits per year. The most frequently visited specialists were physiotherapy (28.6), primary care (15.4), speech therapy (13.1), nursing (10.8), and paediatrics (9.9). Overall, most visits were financed by the SHS. However, a high proportion of visits to physiotherapy (64.2%), speech therapy (44.6%), and psychology (77.5%) were out-of-pocket (Table 2).

**Table 2:** Mean number of visits per patient/year to the different specialist

Visits to professionals	Financed by the SHS	Private insurance	Out-of-pocket	Total
Physiotherapy	8.78	1.43	18.34	28.55
Primary care	10.89	0.51	4.00	15.40
Speech therapy	6.67	0.58	5.83	13.08
Nursing	10.69	0.10	0.00	10.79
Paediatrics	8.95	0.31	0.58	9.85
Occupational therapy	4.90	0.71	2.50	8.10
Psychology	0.55	0.59	3.90	5.03
Neurology	1.77	0.21	0.05	2.02
Odontology	0.92	0.07	0.47	1.45
Cardiology	1.34	0.07	0.03	1.44
Traumatology	1.09	0.06	0.06	1.21
Otorhinolaryngology	1.13	0.05	0.00	1.17
Ophthalmology	1.03	0.03	0.06	1.12
Rehabilitation	0.93	0.00	0.17	1.10
Internal medicine	1.05	0.00	0.00	1.05
Pneumology	0.84	0.05	0.00	0.88
Nutrition	0.64	0.01	0.16	0.81
Neurosurgery	0.76	0.00	0.00	0.76
Endocrinology	0.65	0.02	0.01	0.69
Psychiatry	0.60	0.00	0.03	0.64
Nephrology	0.52	0.00	0.00	0.52
Gastroenterology	0.42	0.02	0.03	0.48
Podiatry	0.29	0.00	0.15	0.44
Palliatives	0.37	0.00	0.00	0.37
Clinical genetics	0.31	0.00	0.00	0.31
Dermatology	0.14	0.09	0.00	0.23
Rheumatology	0.15	0.00	0.00	0.15
Pain unit	0.03	0.00	0.10	0.14
Total	66.41	4.93	36.48	107.81

SHS: Spanish Health System

In addition, 40.1% of the patients had to visit an emergency department during the last year (mean [SD] of 2.0 [3.0] visits), while 41.9% of patients required hospitalization, with a mean (SD) length of stay of 11.5 (30.5) days.

#### Diagnostic tests

According to the HCPs in the study, biochemical, imaging, and other complementary tests are performed for the diagnosis of LSD and during patients’ follow-up visits. Estimation of healthcare resource utilization can be found in Supplementary Table S2.

#### Treatments and surgery

More than half (*n* = 52; 60.5%) of patients received some type of specific therapy for their disease, enzymatic replacement treatment being the most common (*n* = 41; 47.7%), followed by therapy with small molecules (*n* = 4; 4.7%), haematopoietic stem cell transplantation (*n* = 4; 4.7%), and gene therapy (*n* = 3; 3.5%).

On the other hand, during the last three years, 66.9% of patients in the study required at least one surgical intervention, traumatological surgery (*n* = 17; 16.5%) and neurosurgery (*n* = 9; 8.7%) being the most common. Patients also required digestive (*n* = 6; 5.8%), maxillofacial (*n* = 5; 4.9%), ophthalmologic (*n* = 3; 2.9%), orthopaedic (*n* = 2; 1.9%) and cardiovascular surgery (*n* = 2; 1.9%), and bone marrow transplantation (*n* = 1; 1.0%).

### Economic costs

The total estimated cost per patient/year was €228,232.60. Of this, €185,715.70 (81.4%) correspond to direct costs for the SHS, while €36,346.72 (15.9%) and €6,170.20 (2.7%) correspond to indirect costs and direct costs for patients, respectively.

#### Direct costs

Of all direct costs for the SHS, 91.0% correspond to treatment costs, followed, to a much lesser extent, by costs related to hospitalization (3.2%) and visits to specialists (2.4%) (Table 3).

**Table 3:** Distribution of direct costs per patient/year for the SHS

	Average cost for the SHS per service (€)	Percentage of total cost (%)
Treatments	168,919.72	90.96
Hospitalization	5,929.20	3.19
Visits to Primary Care, Nursing, and specialists	4,465.42	2.40
Formal care	1,661.49	0.89
Annual follow-up	1,511.96	0.81
Surgery	1,329.76	0.72
Medical equipment and adaptations	1,171.90	0.63
Visits to the emergency department	561.66	0.30
Diagnostic tests	164.60	0.09
Total	185,715.70	100

SHS: Spanish Health System

#### Indirect costs

Indirect costs per patient/year were estimated at €36,346.72. Of these, 89.0% corresponded to informal care, while the remaining 11.0% corresponded to hours of work lost due to the LSD (Table 4).

**Table 4:** Average indirect costs per patient/year

	Avg. indirect costs per patient/year (€)
**Hours of work lost due to LSD**	3,985.99
Unemployed due to LSD	2,029.91
Sick leave due to LSD	241.08
Reduced working day	1,714.99
**Unpaid care hours**	32,360.73
**Total**	36,346.72

LSD: lysosomal storage disorder

#### Costs for the family unit

The mean annual direct cost for each family unit was €6,170.20. Of these, formal care and out-of-pocket pharmacological treatment accounted for more than half of cost (Table 5).

**Table 5:** Average costs for the family unit/year

	Avg. cost for the family unit/year (€)
Out-of-pocket formal care	2,364.42
Treatments/Rehabilitation/Visits	978.11
Medical equipment	478.94
Home and vehicle adaptation	568.34
Pharmacological treatments	1,254.51
Dietary supplements	512.92
Private insurance	12.95
Total	6,170.20

## Discussion

In this cross-sectional study, we used a questionnaire to describe the situation of 86 Spanish patients with LSDs and their caregivers (*n* = 64), and to estimate the costs associated with these disorders from the perspective of the SHS and patients.

Most patients in our study sample presented some type of limitation and required help from a caregiver, who was their mother in most cases. In this regard, it is worth noting that in our study a higher percentage of women than men were unemployed due to their caregiver role. This is in line with previously published studies reporting data on patients with LSDs showing that these patients require help from caregivers (usually their mothers) whose employment status can be affected by their caregiver role [10, 11, 28].

Patients in our study reported that the disease had a significant impact on different domains, especially in the leisure, mobility, and daily activities domains. In addition, almost all patients reported having mood swings, including anxiety and depression. This result mirrors those obtained in previous surveys performed among patients with LSDs in several European countries which reported that these patients presented physical activity restrictions, with an impact on social and family life as well as the emotional domain, reporting pain and anxiety/depression [9, 10, 29].

Similarly, almost all caregivers who responded to the questionnaire reported an emotional and physical burden with a profound impact on their lives, as previously reported in studies with caregivers of patients with LSDs [11, 30] and other rare diseases [31].

Overall, the results of our study highlight the importance of providing emotional and psychological support to both patients with LSDs and their caregivers. In this context, it is worth noting that most visits to psychology were out-of-pocket, imposing an economic burden on patients or family. Thus, these results showed that patients with LSDs face unmet needs not covered by the SHS.

Regarding healthcare resource utilization, approximately 42% (*n* = 38) of patients with LSDs required hospitalizations with a mean length of 11.5 days, as well as a high number of visits to different specialists (mean of 66.4 visits) per year. These results are similar to those reported in an international study on metachromatic leukodystrophy in which patients required a mean length of inpatient hospital stays of 10.7 to 15.9 days and a mean number of outpatient visits ranging from 5.8 to 62.5 during the last year [11]. However, hospitalization time was slightly higher than that reported in the study by Darbà et al. (mean of 8 days) [32]. Altogether, these results show that the daily management of LSDs can be complex, requiring multiple medical visits and constant care that could be beyond the organizational and physical capacity of families.

This complexity is also reflected in the high rates of hospitalizations and emergency visits observed in our study. These findings must be interpreted in light of the fact that approximately 40% of patients were not receiving any disease-specific therapy. Moreover, even among those undergoing treatment, current interventions may not fully prevent the progression of disease or the occurrence of acute complications.

LSDs impose a great economic burden with a mean annual total cost per patient of approximately €127,000, treatment costs accounting for approximately half of this amount. This result is in line with that reported for Spanish patients in a European study in which the average annual total cost per patient in 2012 was €94,385, approximately a third of these costs corresponding to drugs [10]. This same study reported heterogenous data from other European countries, ranging from €24,520 (Hungary) to €209,420 (Germany). Additionally, the mean hospitalization cost per patient/year in our study was €5,929.20, consistent with a recent Spanish study that reported a mean cost per patient of €5,686 [32].

Families are also economically affected by the LSDs with a mean annual direct cost of approximately €6,000. Approximately a third of the costs assumed by the patient/family correspond to out-of-pocket formal care. Furthermore, patients require the use of out-of-pocket medical equipment and home and vehicle adaptation. Noteworthy is that this is the cost after receiving financial support from the SHS, which means families need to face a much higher original sum. In this context, it is important to mention that costs for families account for approximately 25% of their weighted mean annual income, reported to be €26,032. In addition, almost 30% of caregivers were unemployed due to the LSD, reducing the familiar income and increasing the economic burden. Data in the literature about costs for families are scarce. In this regard, a recent publication about the economic burden of rare diseases in the US showed that non-medical costs, such as spending on home or motor vehicle modifications, per patient in 2019 amounted to $12,310 for children and $4,007 for adults with rare diseases [33].

This study has some limitations. First, given the nature of the study, estimations collected from patients and HCPs might not correspond with data on registries or clinical records. Second, as patients were invited to participate by the Spanish MPS-Lisosomales Association, those outside this association could not be included in the study. In this regard, and considering the low prevalence of the individual disorders, not all LSDs were represented in the study sample, which could influence the costs and impact on QoL estimations. Third, the questionnaire might not cover all aspects of how LSDs impact on patients’ lives. Fourth, the patients were analysed as a global cohort without accounting for potential outcome variations according to the specific type of LSD or impairment (e.g., functional, neurological, visual), or based on treatment status or treatment duration. In this context, although data on current therapy status were collected, the duration of treatment exposure was not systematically recorded, which limits the ability to assess differences related to treatment history. This could result in unaddressed heterogeneity in the data.

Despite the stated limitations, a great number of patients and caregivers from almost all Spanish autonomous communities participated in study. In addition, the study considered the perspective of different specialties involved in the management of the disease.

Future studies should explore how access to treatment may influence non-treatment healthcare costs and indirect costs in patients with LSDs. This line of inquiry would benefit from a longitudinal approach and from analyses stratified by specific diseases or comparable clinical profiles.

In conclusion, our study brings valuable information on LSD-associated costs, highlighting the importance of a comprehensive approach to these disorders in order to guarantee proper medical attention and to reduce the associated costs and the impact these disorders have on patients’ and caregivers’ QoL.

## Electronic supplementary material

Below is the link to the electronic supplementary material.

Supplementary Material 1

## Data Availability

The datasets used and/or analysed during the current study are available from the corresponding author on reasonable request.

## Abbreviations

LSDs: Lysosomal storage disorders
QoL: Quality of life
SHS: Spanish Health System
HCPs: Healthcare professionals
INE: Spanish Statistical Office
SD: Standard deviation
