# Supplementary material for: Human, economic, and social impact of lysosomal storage diseases
Source: Orphanet J Rare Dis. 2025 Nov 25;20:605. doi: 10.1186/s13023-025-04053-z (PMC12645673; doi:10.1186/s13023-025-04053-z)
Supplement: Supplementary file 1 — Supplementary Material 1 [file 13023_2025_4053_MOESM1_ESM.docx]

**Note to the reader:**
This is a direct English translation of the original questionnaire, which was administered in Spanish.
The translation was prepared specifically for this manuscript using artificial intelligence and subsequently reviewed by a researcher to ensure accuracy and fidelity to the original content.

**CRD – PARTICIPANT PROFILE**

**1. Please indicate whether you are the patient or a family member/caregiver.**
A. Patient
B. Family member / caregiver

**CRD – PATIENTS (If P1 “A”)**

**2. Gender: (Single choice)**
A. Male
B. Female

**3. Age: (Enter value in years)**

**4. Autonomous Community of residence: (Single choice from dropdown list)**

**5. Current employment or academic status: (Single choice)**
A. Student
B. Self-employed
C. Employed
D. Unemployed (for reasons unrelated to the disease)
E. Unemployed (due to the disease)
F. Retired (for reasons unrelated to the disease)
G. Retired (due to the disease)

**6. If P5 “B or C”: How many hours per week do you work? (Numeric, 1–60 h/week)**

**7. If P6 < 36h: Type of reduced working hours (Single choice)**
A. Reduced schedule without losing social security contribution
B. Reduced schedule with reduction in social security contribution

**8. If P6 < 36h: Was the schedule reduction due to the disease? (Single choice)**
A. Yes
B. No

**9. If P5 “B, C, D, E, F, G”: Have you been on medical leave in the past 12 months? (Single choice)**
A. Yes
B. No

**10. If P9 “A”: Approximate total duration of leave in the past 12 months (Numeric, in days)**

**11. What is your officially recognized level of disability? (Single choice)**
A. I don’t have a recognized disability
B. Less than 33%
C. 33–64%
D. 65% or more

**12. Do you have a caregiver for certain tasks? (Multiple choice, except “E”)**
A. Yes, a family member
B. Yes, a professional funded by the National Health System (free of charge to the patient)
C. Yes, a school assistant funded by the education system (free of charge to the patient)
D. Yes, a non-funded professional (cost borne by the patient/family)
E. No

**13. If P12 “A”: What is your relationship with the caregiver? (Multiple choice)**
A. Father
B. Mother
C. Brother
D. Sister
E. Son/Daughter
F. Other

**14. If P12 “A”: How many hours of care per day do you receive from your family member? (Numeric, 1–24 h/day)**

**15. If P12 “B”: How many hours of care per day do you receive from the publicly funded professional?**

**16. If P12 “C”: How many hours of care per day do you receive from the school-funded professional?**

**17. If P12 “D”: How many hours of care per day do you receive from the non-funded caregiver?**

**18. Type of lysosomal disease: (Single choice from dropdown list)**

**19. When were you diagnosed with the disease? (Single choice)**
A. Before birth
B. Before turning 1 year old
C. After turning 1 year old

**20. If P19 “B”: How many months old were you when you were diagnosed? (Numeric, 0–12)**

**21. If P19 “C”: How many years old were you when you were diagnosed? (Numeric, 1–99)**

**22. If P19 “B or C”: How long after the first symptoms did the diagnosis occur? (If less than 1 year, write 0)**

**23. Have you received or are you currently receiving specific treatment for the disease? (Multiple choice, except A)**
A. No
B. Yes, enzyme replacement therapy
C. Yes, small molecule therapy
D. Yes, gene therapy
E. Yes, transplant

**24. If P23 “B, C, D or E”: Time from diagnosis to start of first treatment (Enter value in years; if less than one year, enter 0)**

**25. If P23 “B”: Where are you currently receiving enzyme replacement therapy? (Single choice)**
A. In a hospital
B. At home

**26. What is your current degree of physical/mobility limitation? (Single choice)**
A. None
B. Mild (I walk and am independent, but need help with certain tasks. I use a cane/wheelchair only for long distances)
C. Moderate (I need help for daily tasks, I can walk with assistive devices such as a cane or crutch)
D. Severe (I do not walk, but can do transfers or walk a few meters with assistive devices)
E. Total
F. I don’t know

**27. What is your current level of visual impairment? (Single choice)**
A. None
B. Mild (corrected with lenses)
C. Moderate (I can perform visual tasks with inaccuracies, requiring extra time and adaptations)
D. Severe (I struggle with gross visual tasks; unable to perform detailed visual tasks)
E. Blindness (no vision or only light perception; unable to perform visual tasks)
F. I don’t know

**28. What is your current level of hearing impairment? (Single choice)**
A. None
B. Mild (I can hear some speech but not whispers clearly)
C. Moderate (I struggle to hear someone speaking at a normal volume)
D. Severe (I cannot hear normal conversation and can only detect loud sounds)
E. Profound (I hear nothing)
F. I don’t know

**29. On a scale from 0 to 10 (0 = worst imaginable health, 10 = best imaginable health), how would you rate your overall health during the past month?**

**30. During the past month, which of the following moods have you experienced? (Multiple choice, except “I”)**
A. Anxiety
B. Depression
C. Irritability
D. Apathy
E. Guilt
F. Insecurity
G. Helplessness
H. Confusion
I. None of the above

**31. On a scale from 0 to 10 (0 = no pain; <3 = mild; 3–5 = moderate; 6–8 = intense; 9–10 = unbearable), how would you rate your pain in the past month?**

**32. How much does your disease affect you in the following areas? (0 = not at all, 10 = totally)**
Functional/mobility: _____
Daily activities: _____
Emotional state: _____
Social relationships: _____
Intimate relationships: _____
Academic/professional life: _____
Leisure/sports: _____

**33. Approximately, what is your household’s total monthly income, including benefits? (Single choice)**
A. Less than €1,260
B. €1,260–€1,600
C. €1,600–€2,000
D. €2,000–€3,000
E. More than €3,000
F. Prefer not to answer

**34. Do you have difficulty making ends meet? (Single choice)**
A. Yes
B. No

**35. Do you have private health insurance? (Single choice)**
A. Yes
B. No

**36. If P35 “A”: Approximately, how much do you pay monthly for your private insurance (excluding co-pays or extra costs)?**
_______ €/month

**37. Please select the healthcare professionals you have visited in the past year due to your disease. (Multiple choice except “Z”)**
[List identical to CRD familiar, omit for brevity]

**38. For each professional selected in P37: How many visits related to your disease did you have in the past year?**

- Visits covered by the National Health System: _____
- Private visits covered by insurance: _____
- Private visits paid directly by patient/family: _____

**39. How many emergency visits related to your disease did you have in the past year? (If none, write “0”)**

**40. How many days have you been hospitalized due to your disease in the past year? (If none, write “0”)**

**41. What types of surgeries related to your disease have you undergone in the past 3 years? (Multiple choice except “A”)**
A. None
B. Bone marrow transplant
C. Neurosurgery
D. Cardiac or cardiovascular surgery
E. Digestive system surgery
F. Kidney surgery
G. Maxillofacial surgery
H. Orthopedic surgery
I. Traumatological surgery
J. Eye surgery
K. Other

**42. In the past year, what was the approximate out-of-pocket cost of medications related to your disease?**
_______ €

**43. In the past year, what was the approximate cost of dietary supplements, nutritional products, and hygiene items related to the disease?**
_______ €

**44. Select the types of transportation used for medical visits in the past year (Multiple choice). For each, indicate if it was:**
A. Funded (no cost to the patient)
B. Not funded (paid by patient)

Options: Car/Taxi, Metro/Bus/Local train, Train, Ambulance, Plane, Boat/Ferry

**45. Approximately how long does it take to get from your home to the healthcare center? (One-way, in minutes, 1–600)**

**46. On average, how long do your visits last, including waiting time? (In minutes, 1–600)**

**47. What types of medical devices or health products have you used during your disease? (Multiple choice except “R”)**
[List identical to CRD familiar]

**48. If P47 ≠ “R”: Approximately, what amount have you paid in the last 5 years for the devices/products listed above? (If none, enter 0)**
_______ €

**49. Have you made any home or vehicle adaptations due to the disease? (Single choice)**
A. Yes
B. No

**50. If P49 “A”: What was the cost of those adaptations?**
_______ €

**51. If P48 or P50 > 0 €: Have you received any subsidies or financial aid to cover the cost of devices/adaptations? (Single choice)**
A. Yes
B. No

**52. If P51 “A”: Approximately what percentage of the total cost was covered by the subsidy/aid? (Numeric, 1–100%)**
_______ %

**CRD – FAMILY MEMBERS / CAREGIVERS (If P1 “A”)**

**2. Patient’s gender: (Single choice)**
A. Male
B. Female

**3. Patient’s age: (Numeric, 0–99 years)**

**4. Autonomous Community of residence of the patient: (Single choice from dropdown list)**

**5. Patient’s current academic or work situation: (Single choice)**
A. Not enrolled in school
B. Student
C. Self-employed
D. Employed
E. Unemployed (for reasons unrelated to the disease)
F. Unemployed (due to the disease)
G. Retired (for reasons unrelated to the disease)
H. Retired (due to the disease)

**6. If P5 “C or D”: How many hours per week does the patient work? (Numeric, 1–60 h/week)**

**7. If P6 < 40h: Type of reduced working hours (Single choice)**
A. Reduced schedule without losing social security contribution
B. Reduced schedule with reduction in social security contribution

**8. If P6 < 40h: Was the schedule reduction due to the disease? (Single choice)**
A. Yes
B. No

**9. If P5 “C, D, E, F, G or H”: Has the patient been on medical leave in the past 12 months? (Single choice)**
A. Yes
B. No

**10. If P20 “A”: Approximate total duration of the patient’s leave in the past 12 months (Numeric, in days)**

**11. What is the patient’s officially recognized level of disability? (Single choice)**
A. No recognized disability
B. Less than 33%
C. 33–64%
D. 65% or more

**12. What is your relationship to the patient? (Single choice)**
A. Son/Daughter
B. Sibling
C. Parent
D. Other family relationship
E. In-law
F. No family relationship

**13. How many hours per day do you dedicate to caring for the patient? (Numeric, 1–24 h/day)**

**14. Your gender: (Single choice)**
A. Male
B. Female

**15. Your age: (Numeric, 14–99 years)**

**16. Your current employment status: (Single choice)**
A. Student
B. Self-employed
C. Employed
D. Unemployed (for reasons unrelated to caregiving)
E. Unemployed (due to your caregiving role)
F. Retired (for reasons unrelated to caregiving)
G. Retired (due to caregiving; e.g., early retirement to care for the patient)

**17. If P16 “B or C”: Weekly working hours (Numeric, 1–60)**

**18. If P17 < 36h: Type of reduced working hours (Single choice)**
A. Reduced schedule without losing social security contribution
B. Reduced schedule with reduction in social security contribution

**19. If P17 < 36h: Was the schedule reduction due to your caregiving role? (Single choice)**
A. Yes
B. No

**20. If P16 “C, D, E, F, G or H”: Have you been on leave in the past 12 months? (Single choice)**
A. Yes
B. No

**21. If P20 “A”: Total duration of your leave in the past 12 months (Numeric, in days)**

**22. Besides you, does the patient have another caregiver? (Multiple choice except “E”)**
A. Yes, another family member
B. Yes, a professional funded by the National Health System (free of charge to the patient)
C. Yes, a school assistant funded by the education system (free of charge to the patient)
D. Yes, a non-funded professional (cost borne by the patient/family)
E. No

**23. If P22 “A”: How many hours of care per day does the patient receive from the other family caregiver? (Numeric, 1–24)**

**24. If P22 “B”: How many hours of care per day does the patient receive from the public healthcare professional? (Numeric, 1–24)**

**25. If P22 “C”: How many hours of care per day does the patient receive from the school-funded professional? (Numeric, 1–24)**

**26. If P22 “D”: How many hours of care per day does the patient receive from the non-funded caregiver? (Numeric, 1–24)**

**27. Type of lysosomal disease the patient has: (Single choice from dropdown list)**

**28. When was the patient diagnosed with the disease? (Single choice)**
A. Before birth
B. Before turning 1 year old
C. After turning 1 year old

**29. If P28 “B”: How many months old was the patient at diagnosis? (Numeric, 0–12)**

**30. If P28 “C”: How many years old was the patient at diagnosis? (Numeric, 1–80)**

**31. If P28 “B or C”: How much time passed between the first symptoms and the diagnosis? (If less than 1 year, enter 0) (Numeric, 0–50 years)**

**32. Has the patient received or is currently receiving specific treatment for the disease? (Multiple choice except “A”)**
A. No
B. Yes, enzyme replacement therapy
C. Yes, small molecule therapy
D. Yes, gene therapy
E. Yes, transplant

**33. If P32 “B, C, D or E”: How much time passed between diagnosis and the start of the first treatment? (If less than 1 year, enter 0) (Numeric, 0–50 years)**

**34. If P32 “B”: Where is the patient currently receiving enzyme replacement therapy? (Single choice)**
A. In a hospital
B. At home

**35. In your opinion, what is the patient’s current level of cognitive impairment? (Single choice)**
A. None
B. Mild
C. Moderate
D. Severe
E. Profound
F. I don’t know

**36. In your opinion, what is the patient’s current level of physical/mobility limitation? (Single choice)**
A. None
B. Mild
C. Moderate
D. Severe
E. Total
F. I don’t know

**37. In your opinion, what is the patient’s current level of visual impairment? (Single choice)**
A. None
B. Mild (corrected with lenses)
C. Moderate
D. Severe
E. Blindness
F. I don’t know

**38. In your opinion, what is the patient’s current level of hearing impairment? (Single choice)**
A. None
B. Mild
C. Moderate
D. Severe
E. Profound
F. I don’t know

**39. On a scale from 0 to 10 (0 = worst imaginable health, 10 = best imaginable health), how would you rate the patient’s overall health during the past month?**

**40. How much does the disease affect the patient in the following areas? (0 = not at all, 10 = totally)**
Functional/mobility: _____
Daily activities: _____
Emotional state: _____
Social relationships: _____
Academic/professional life: _____
Leisure/sports: _____

**41. On a scale from 0 to 10 (0 = not at all, 10 = totally): During the past month, how much has your caregiving role impacted your own daily activities?**

**42. During the past month, which of the following moods have you experienced? (Multiple choice except “I”)**
A. Anxiety
B. Depression
C. Irritability
D. Apathy
E. Guilt
F. Insecurity
G. Helplessness
H. Confusion
I. None of the above

**43. How much has your caregiving role affected you in the following areas? (0 = not at all, 10 = totally)**
Functional/mobility: _____
Daily activities: _____
Emotional state: _____
Social relationships: _____
Academic/professional life: _____
Leisure/sports: _____

**44. Approximately, what is your household’s total monthly income, including benefits? (Single choice)**
A. Less than €1,260
B. €1,260–€1,600
C. €1,600–€2,000
D. €2,000–€3,000
E. More than €3,000
F. Prefer not to answer

**45. Do you have difficulty making ends meet? (Single choice)**
A. Yes
B. No

**46. Does the patient have private health insurance? (Single choice)**
A. Yes
B. No

**47. If P46 “A”: Approximately, how much do you pay monthly for the patient’s private insurance (excluding co-pays or additional costs)?**
_______ €/month

**48. Please indicate the healthcare professionals the patient has visited in the past year due to their disease. (Multiple choice except “Z”)**
A. Primary care physician
B. Primary care pediatrician
C. Hospital pediatrician
D. Cardiologist
E. Dermatologist
F. Endocrinologist
G. Nutritionist
H. Gastroenterologist
I. Nephrologist
J. Neurologist
K. Neurosurgeon
L. Pulmonologist
M. Clinical geneticist
N. Rheumatologist
O. Dentist
P. Ophthalmologist
Q. ENT specialist
R. Speech therapist
S. Physiotherapist
T. Rehabilitation specialist
U. Podiatrist
V. Orthopedic surgeon
W. Psychologist
X. Psychiatrist
Y. Internist
Z. None of the above
AA. Pain unit
AB. Occupational therapist
AC. Palliative care specialist
AD. Nurse

**49. For each professional selected in P48: Approximately how many visits related to the disease did the patient have in the past year?**

- Visits funded by the National Health System: _____
- Private visits covered by insurance: _____
- Private visits paid directly by patient/family: _____

**50. How many emergency visits related to the patient’s disease did they have in the past year? (If none, enter 0)**

**51. How many days was the patient hospitalized due to the disease in the past year? (If none, enter 0)**

**52. What types of surgeries related to the disease has the patient undergone in the past 3 years? (Multiple choice except “A”)**
A. None
B. Bone marrow transplant
C. Neurosurgery
D. Cardiac or cardiovascular surgery
E. Digestive system surgery
F. Kidney surgery
G. Maxillofacial surgery
H. Orthopedic surgery
I. Traumatological surgery
J. Eye surgery
K. Other

**53. In the past year, what was the patient’s approximate out-of-pocket cost for medications related to the disease?**
_______ €

**54. In the past year, what was the approximate cost of dietary supplements, nutritional products, and hygiene products related to the disease?**
_______ €

**55. Select the types of transportation used in the past year for the patient’s medical visits (Multiple choice). For each, specify if it was funded or not:**
Options:

1. Car/Taxi
2. Metro/Train (e.g., commuter rail)/Bus
3. Long-distance train
4. Ambulance
5. Plane
6. Boat/Ferry

Funded: A
Not funded (out-of-pocket): B

**56. How often do you accompany the patient to medical visits? (Single choice)**
A. Always
B. 75% of the time
C. 50% of the time
D. 25% of the time
E. Never

**57. Approximately how long does it take (one way) to travel from your home to the patient’s healthcare center? (Numeric, 1–600 min)**

**58. On average, how long do medical visits last, including waiting time? (Numeric, 1–600 min)**

**59. What types of medical devices or health products has the patient used in the past year? (Multiple choice except “R”)**
A. Crutches
B. Manual wheelchair
C. Electric wheelchair
D. Corsets
E. Special cushions/belts
F. Prosthetics
G. Hearing aids
H. Glasses
I. Cochlear implants
J. Non-invasive home ventilation
K. Oxygen therapy
L. Adapted oral nutrition
M. Enteral home nutrition
N. Electric blankets
O. Massagers/muscle stimulators
P. Eye-related products (e.g., glasses, lenses)
Q. Other
R. None

**60. If P59 ≠ “R”: Approximately, how much have you paid or financed in the past 5 years for the above products?** (If none, enter 0)
_______ €

**61. Have you had to make any adaptations to your home or vehicle due to the patient’s disease? (e.g., removing steps, installing ramps, widening doors, changing vehicles) (Single choice)**
A. Yes
B. No

**62. If P61 “A”: What was the cost of the home or vehicle adaptations?**
_______ €

**63. If P60 or P62 > 0 €: Have you received financial aid or subsidies to partially or fully cover the costs of medical devices or home adaptations? (Single choice)**
A. Yes
B. No

**64. If P63 “A”: Approximately what percentage of the total cost was covered by aid/subsidies? (Numeric, 1–100%)**
_______ %
